# Stiefel-Whitney topological charges in a three-dimensional acoustic nodal-line crystal

**DOI:** 10.1038/s41467-023-40252-7

**Published:** 2023-07-28

**Authors:** Haoran Xue, Z. Y. Chen, Zheyu Cheng, J. X. Dai, Yang Long, Y. X. Zhao, Baile Zhang

**Affiliations:** 1 Division of Physics and Applied Physics, School of Physical and Mathematical Sciences, Nanyang Technological University, Singapore, Singapore; 2 National Laboratory of Solid State Microstructures and Department of Physics, Nanjing University, Nanjing, China; 3 Department of Physics and HKU-UCAS Joint Institute for Theoretical and Computational Physics at Hong Kong, The University of Hong Kong, Hong Kong, China; 4 HK Institute of Quantum Science & Technology, The University of Hong Kong, Hong Kong, China; 5 Centre for Disruptive Photonic Technologies, Nanyang Technological University, Singapore, Singapore

**Keywords:** Acoustics, Topological matter

## Abstract

Band topology of materials describes the extent Bloch wavefunctions are twisted in momentum space. Such descriptions rely on a set of topological invariants, generally referred to as topological charges, which form a characteristic class in the mathematical structure of fiber bundles associated with the Bloch wavefunctions. For example, the celebrated Chern number and its variants belong to the Chern class, characterizing topological charges for complex Bloch wavefunctions. Nevertheless, under the space-time inversion symmetry, Bloch wavefunctions can be purely real in the entire momentum space; consequently, their topological classification does not fall into the Chern class, but requires another characteristic class known as the Stiefel-Whitney class. Here, in a three-dimensional acoustic crystal, we demonstrate a topological nodal-line semimetal that is characterized by a doublet of topological charges, the first and second Stiefel-Whitney numbers, simultaneously. Such a doubly charged nodal line gives rise to a doubled bulk-boundary correspondence—while the first Stiefel-Whitney number induces ordinary drumhead states of the nodal line, the second Stiefel-Whitney number supports hinge Fermi arc states at odd inversion-related pairs of hinges. These results experimentally validate the two Stiefel-Whitney topological charges and demonstrate their unique bulk-boundary correspondence in a physical system.

## Abstract (web summary)

Symmetry plays a crucial role in defining the band topology. Here, the authors experimentally demonstrate that spacetime inversion symmetry can lead to Stiefel-Whitney topological charges and protect hinge states in an acoustic nodal-line semimetal.

## Introduction

Quantum mechanical wavefunctions are written in complex numbers, and so are the Bloch wavefunctions in crystals. These complex Bloch wavefunctions are twisted in momentum space to form band topology, following their mathematical structure of fiber bundles that is characterized by a set of topological invariants corresponding to a characteristic class. A famous example of the topological invariant is the Chern number in the Chern class, which can be treated as a topological charge that induces topological boundary states, following the principle of bulk-boundary correspondence^1^. Such a correspondence from bulk to boundary is generally one to one, since different topological phases are incompatible and do not exist simultaneously to host different topological charges. Materials classified in the Chern class have been extensively explored for decades, leading to many discoveries such as the Chern insulators, time-reversal-invariant topological insulators, and Weyl semimetals^2–7^.

In the presence of symmetries, the properties of the Hamiltonian eigenspace can be significantly modified^8^. A prominent example is the spacetime inversion (*PT*) symmetry. In the field of non-Hermitian physics, *PT* symmetry has played a central role as it can lead to real eigenenergies that are unexpected for a non-Hermitian Hamiltonian^9^. In periodic Hermitian systems without spin-orbit coupling, while the eigenenergies are already real, the application of *PT* symmetry is able to refine the Bloch wavefunctions from complex numbers to real numbers^10,11^. Accordingly, the Chern number must vanish in such a scenario, and the Chern class classification is no longer applicable. Instead, the Stiefel-Whitney (SW) class is responsible for the topological classification of the *PT*-symmetric systems with purely real eigenspaces^12^.

The SW class consists of two topological charges, the first and second SW numbers, classifying 1D and 2D *PT*-symmetric systems, respectively. A nontrivial first (second) SW number represents the obstruction of finding a global real basis of fiber bundles for Bloch wavefunctions in the 1D (2D) Brillouin zone^12^. This context is similar to the Chern number in the obstruction of finding a global complex basis for Bloch wavefunctions in the 2D Brillouin zone. While the first SW number is equivalent to the quantized Berry phase^13,14^, the second SW number is unique to the SW class, being able to protect 2D higher-order topological insulators and 3D topological semimetals^15–23^, as counterparts of Chern insulators and Weyl semimetals protected by the Chern number. More intriguingly, recent theories suggest that nontrivial first and second SW numbers can co-exist in a single system^17,18,20,21,23^, leading to a doubled bulk-boundary correspondence—the same bulk can be doubly charged with two topological charges simultaneously, which give rise to two kinds of boundary states at different locations.

**Fig. 1:**
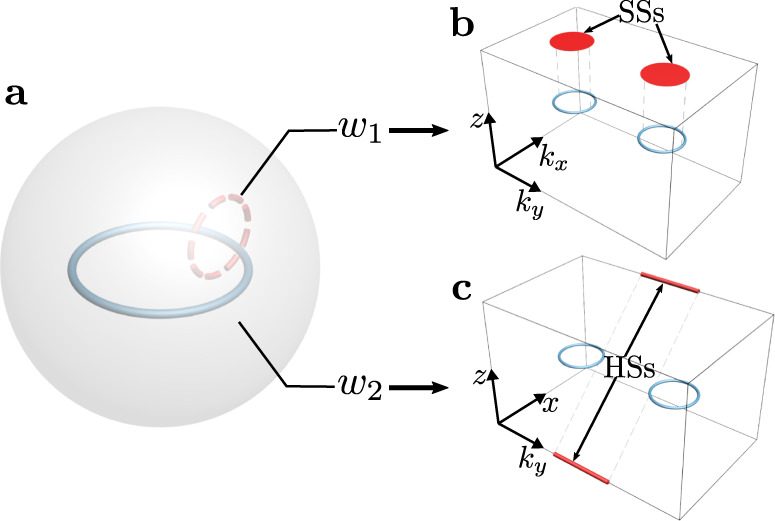
Doubly charged nodal line. **a** Illustration of a nodal line with a doublet of topological charges (*w*_1_, *w*_2_). *w*_1_ and *w*_2_ are defined on the chosen *S*^1^ and *S*^2^ surrounding the nodal line, respectively. **b** Due to *w*_1_ = 1, the surface states (SSs) form drumheads bounded by the projections of bulk nodal lines on the surface Brillouin zone. **c** *w*_2_ = 1 leads to a *PT*-related pair of hinge state (HS) Fermi arcs bounded by the projections of the bulk nodal lines on the hinge Brillouin zone.

Here, in a three-dimensional (3D) acoustic crystal, we experimentally realize a nodal-line topological semimetal with a doublet of SW topological charges as illustrated in Fig. 1a, with *w*_1_ and *w*_2_ the first and second SW numbers, respectively (Supplementary Section 1 and Supplementary Fig. S1). Such a nodal line can be named a real nodal line due to its purely real eigenspace. Because of the nontrivial *w*_2_, these nodal lines appear in pairs (see Fig. 1b, c)^17,21^, resembling the Nielsen-Ninomiya theorem of Weyl points in Weyl semimetals^24^. The 1D topological charge *w*_1_ leads to the first-order drumhead surface states (SSs)^25^, which also appear in the case of a conventional nodal line (see Fig. 1b). However, the additional 2D topological charge *w*_2_, which is unique to the SW class, can give rise to odd *PT*-related pairs of hinge Fermi arcs. In our experiment, this is demonstrated by a sample of a long rectangular prism that hosts a single pair of *PT*-related gapless hinges (see Fig. 1c). The novel distribution of hinge states (HSs) distinguishes this unconventional nodal-line semimetal from other existing second-order topological semimetals that host HSs on all four hinges^26–28^. This novelty is further experimentally confirmed on a sample with a more irregular but still *PT*-invariant geometry.

## Results

### General idea

Let us start with introducing the minimal Dirac model for a nodal line with a doublet of topological charges (*w*_1_, *w*_2_) (Supplementary section 2 and Supplementary Figs. S2-S4):

1 $${{{{{{{\mathcal{H}}}}}}}}({{{{{{{\bf{k}}}}}}}})={k}_{x}{\gamma }_{1}+{k}_{y}{\gamma }_{2}+{k}_{z}{\gamma }_{3}+{m}_{z}{{{{{{{\rm{i}}}}}}}}{\gamma }_{3}{\gamma }_{4}.$$

**Fig. 2:**
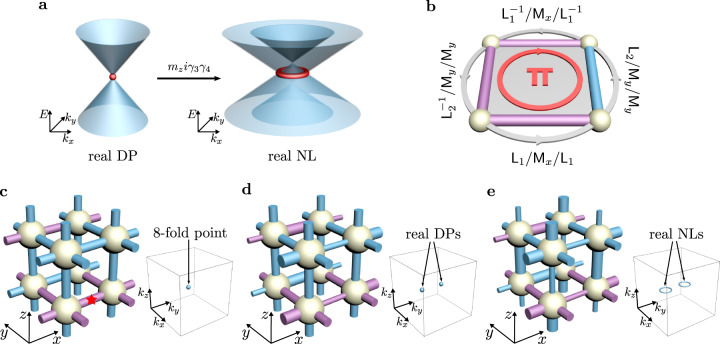
Illustrations for the Dirac model, projective symmetry algebras and the tight-binding model. **a** The fourfold degenerate real Dirac point (DP) is monotonically spread into a real nodal line (NL) by the partial mass term of *m*_*z*_i*γ*_3_*γ*_4_. **b** Successively implementing operators sends a particle to circle the rectangular plaquette with flux *π*. Here, positive (negative) hopping amplitudes are colored in purple (blue). **c** The undimerized lattice with flux *π* through every rectangular plaquette. The red star denotes the inversion center. A topologically neutral eightfold degenerate crossing point resides at *K* = (*π*, *π*, *π*) in the Brillouin zone. **d**, The dimerization ${{\mathfrak{D}}}_{1}$ is added along the *x*-direction, which is alternative along the *y* direction and uniform along the *z*-direction. Hence, the eightfold degenerate crossing point is split into a pair of fourfold degenerate real DPs, each with topological charge *w*_2_ = 1. **e** The dimerization ${{\mathfrak{D}}}_{2}$ along the *z* direction is further added, which alternates along both *x* and *y* directions. Then, each real DP is spread into a real NL.

Here, *γ*_*a*_ with *a* = 1, 2, ⋯  , 5 are the 4 × 4 Hermitian Dirac matrices satisfying the Clifford algebra: {*γ*_*a*_, *γ*_*b*_} = 2*δ*_*ab*_1_4_ and **k** = (*k*_*x*_, *k*_*y*_, *k*_*z*_) is the wavevector. Without loss of generality, we represent the *PT* operator as ${{{{{{{\mathcal{P}}}}}}}}{{{{{{{\mathcal{T}}}}}}}}={{{{{{{\mathcal{K}}}}}}}}$ with ${{{{{{{\mathcal{K}}}}}}}}$ the complex conjugation. In model (1), we have ordered the Dirac matrices so that *γ*_*i*_ with *i* = 1, 2, 3 are real and *γ*_4,5_ are purely imaginary. A set of matrices representing *γ*_*a*_ can be found in Methods. Hence, it is easy to check (1) is indeed a real Hamiltonian preserving *PT* symmetry. Moreover, since *iγ*_3_*γ*_4_ anticommutes with *γ*_3_ while commutes with *γ*_1,2_, we may refer to *m*_*z*_i*γ*_3_*γ*_4_ as the partial mass term along the *k*_*z*_ direction. The nodal line lies on the *k*_*x*_-*k*_*y*_ plane, and its radius increases monotonically as *m*_*z*_ (see Fig. 2a). When *m*_*z*_ = 0, the ring shrinks into a Dirac point named a real Dirac point due to its real eigenspace^11^.

Inspired by this continuum model, we develop a lattice construction method briefly introduced as follows. First, we shall form an eightfold degenerate band crossing point in the momentum space. Although the crossing point is topologically neutral, we then add appropriate symmetry-breaking dimerization patterns in order to split it into two fourfold degenerate real Dirac points, each with topological charge *w*_2_ = 1. The last step is to further spread each point into a nodal line with certain appropriate dimerization patterns.

To achieve a nodal point with high degeneracy, we utilize projective symmetries, which stem from the gauge fluxes on the lattice and can lead to high dimensional irreducible representations^29,30^. Moreover, the ${{\mathbb{Z}}}_{2}$ lattice gauge fields are highly engineerable in artificial lattices (i.e., the sign of each real hopping amplitude can be flexibly tuned to be + or − ; see Fig. 2b), as demonstrated in recently realized projectively symmetry-protected topological phases in acoustic crystals^31,32^.

### Model construction

As aforementioned, to construct a realizable lattice model, we have recourse to the projective symmetry algebra. We consider a 3D rectangular lattice with the nearest-neighbor hoppings, which has flux *π* for each rectangular plaquette along any direction. The flux pattern can be described by numerous configurations of signs of hopping amplitudes, and the one we choose is given in Fig. 2c–e. Because of the gauge fluxes, the unit translation operators L_*i*_ with *i* = *x*, *y*, *z*, which previously mutually commute, become pairwisely anti-commuting, i.e., {L_*i*_, L_*j*_} = 0 for *i* ≠ *j*, which constitute the projective symmetry algebra of translations. The anti-commutation relation manifests the Aharonov-Bohm effect, since the equivalent form ${{\mathsf{L}}}_{j}^{-1}{{\mathsf{L}}}_{i}^{-1}{{\mathsf{L}}}_{j}{{\mathsf{L}}}_{i}=-1$ corresponds to that a particle accumulates a phase factor *e*^*iπ*^ = − 1 after circling the plaquette spanned by L_*i*_ and L_*j*_, as illustrated in Fig. 2b. Similar analysis shows the projective algebraic relations {M_*i*_, M_*j*_} = 2*δ*_*ij*_ for mirror reflections M_*i*_, and those between M_*i*_ and L_*j*_: ${{\mathsf{M}}}_{i}{{\mathsf{L}}}_{i}{{\mathsf{M}}}_{i}={{\mathsf{L}}}_{i}^{-1}$ and {M_*i*_, L_*j*_} = 0 for *i* ≠ *j* (see Fig. 2b). Here, M_*i*_ reverses the *i*th coordinate with the center of the unit cell as the coordinate origin. We now turn to the Brillouin zone, and denote the representations of L_*i*_ and M_*i*_ as ${{{{{{{{\mathcal{L}}}}}}}}}_{i}^{{{{{{{{\bf{k}}}}}}}}}$ and ${{{{{{{{\mathcal{M}}}}}}}}}_{i}$, respectively. Note that ${{{{{{{{\mathcal{L}}}}}}}}}_{i}^{{{{{{{{\bf{k}}}}}}}}}$ depend on **k**, while ${{{{{{{{\mathcal{M}}}}}}}}}_{i}$ are independent of **k**. Specializing at the point *K* = (*π*, *π*, *π*), their projective algebraic relations are given by

2 $$-\left\{{{{{{{{{\mathcal{L}}}}}}}}}_{i}^{K},\, {{{{{{{{\mathcal{L}}}}}}}}}_{j}^{K}\right\} \,=\, \left\{{{{{{{{{\mathcal{M}}}}}}}}}_{i},\, {{{{{{{{\mathcal{M}}}}}}}}}_{j}\right\} \,=\, 2{\delta }_{ij},\quad \left\{{{{{{{{{\mathcal{M}}}}}}}}}_{i},\, {{{{{{{{\mathcal{L}}}}}}}}}_{j}^{K}\right\}=0.$$

Since time reversal *T* is preserved at *K*, we further consider its representation ${{{{{{{\mathcal{T}}}}}}}}$, which commutes with all ${{{{{{{{\mathcal{L}}}}}}}}}_{i}^{K}$ and ${{{{{{{{\mathcal{M}}}}}}}}}_{i}$. The projective symmetry algebra generated by ${{{{{{{{\mathcal{L}}}}}}}}}_{i}^{K}$, ${{{{{{{{\mathcal{M}}}}}}}}}_{i}$ and ${{{{{{{\mathcal{T}}}}}}}}$ is equivalent to the real Clifford algebra ${C}^{3,3}\otimes {C}^{1,1}\cong {C}^{4,4}\cong {\mathbb{R}}({2}^{d+1})$, which has a unique complex irreducible representation with dimension 2^3^. That is, there is the desired eightfold degenerate crossing point at *K* protected by the projective symmetry algebra (2). Since our unit cell consists of 8 sites, all bands are enforced to cross at *K* to represent (2).

To have a nontrivial SW class, we consider the inversion symmetry P centered at the *x*-bond of the unit cell in Fig. 2c (indicated by the red star), i.e., P = L_*y*_L_*z*_M_*x*_M_*y*_M_*z*_. Following the aforementioned method, we should proceed to add P*T*-invariant dimerization patterns that break some L_*i*_ and M_*j*_ to realize the real nodal lines. Let us consider two dimerization patterns in order. The first pattern ${{\mathfrak{D}}}_{1}$ is the dimerization along the *x* direction, which alternates along the *y* direction but is invariant along the *z* direction as illustrated in Fig. 2d. Adding ${{\mathfrak{D}}}_{1}$ splits the eightfold degenerate topologically “neutral” crossing point into two fourfold degenerate real Dirac points charged by *w*_2_, each of which is modeled by (1) with *m*_*z*_ = 0. We then introduce the second dimerization pattern ${{\mathfrak{D}}}_{2}$, which gives rise to the partial mass term *m*_*z*_i*γ*_3_*γ*_4_ of (1) and therefore can spread each real Dirac point into a nodal line. ${{\mathfrak{D}}}_{2}$ is designed as the dimerization along the *z*-direction, which alternates along both the *x* and *y* directions, as illustrated in Fig. 2e. Each of the nodal lines, as shown in Supplementary section 3 and Supplementary Fig. S5, carries a nontrivial doublet of topological charges (*w*_1_, *w*_2_) as expected.

**Fig. 3:**
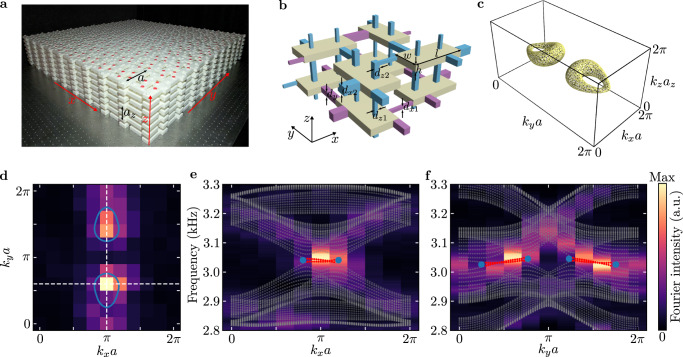
Acoustic crystal design and the observation of drumhead surface states. **a** Experimental sample for measuring the surface states (SSs). The lattice constants in the *xy* plane and along the *z* direction are *a* = 140 mm and *a*_*z*_ = 70 mm, respectively. **b** Unit cell of the acoustic crystal, with tubes enabling positive and negative couplings colored in purple and blue, respectively. The dimensions of the cuboid cavities are *l* = 56 mm, *w* = 28 mm and *h* = 7 mm. The width parameters of the tubes are *d*_*x*1_ = 3.2 mm, *d*_*x*2_ = 7.8 mm, *d*_*y*_ = 6 mm, *d*_*z*1_ = 3.2 mm and *d*_*z*2_ = 4.8 mm. The acoustic crystal is filled with air and surrounded by hard walls. **c** Equi-frequency surface of the acoustic crystal in **b** at 3020 Hz (slightly below the frequency range of the nodal ring) calculated from full-wave simulations. **d** Experimentally measured SS dispersion at 3045 Hz (inside the frequency range of the nodal ring). The solid blue curves denote the projections of the nodal rings. **e**, **f** Experimentally measured SS dispersion along the horizontal (vertical) white dashed line in **d**. The gray and red dots represent simulated eigenfrequencies of the bulk and SSs, respectively. The blue dots denote the projections of the bulk nodal points.

This tight-binding model with *π* fluxes on a simple rectangular lattice can be implemented using the coupled acoustic cavity structure^31–40^. Our designed acoustic crystal is shown in Fig. 3a, with each site in the tight-binding model implemented by a cuboid cavity supporting a dipolar resonance at around 3100 Hz. The coupling between two cavities is enabled by a tube with a square cross-section, with the coupling sign determined by the position of the tube and the coupling amplitude controlled by the width of the tube. Thus, by carefully engineering the coupling tubes, the required gauge fluxes and coupling dimerizations can be realized (see Fig. 3b). The whole structure is filled with air and surrounded by photosensitive resins, which can be treated as rigid walls for sound due to their large impedance mismatch with air (see Methods). Figure 3c shows the equi-frequency surface of the acoustic crystal obtained from full-wave simulation at 3020 Hz (slightly below the minimum frequency of the nodal line), which reveals the existence of two nodal lines forming two rings in the bulk dispersion and suggests the validity of this acoustic design (see Supplementary section 4 and Supplementary Figs. S6-S7 for more details on the dispersion).

### Drumhead surface states

We first demonstrate the existence of conventional drumhead SSs due to the nontrivial first-order topology induced by *ω*_1_. To this end, we scan the acoustic fields on the top surface excited by a speaker placed at the surface center (see Methods and Supplementary Fig. S9). Figure 3d shows the corresponding Fourier intensity at 3045 Hz, where the hot spots occur at positions inside the projections of the nodal rings (denoted by the blue lines), consistent with the feature of the drumhead SSs. To further confirm the existence of the drumhead SSs, we also plot in Fig. 3e, f the frequency-resolved Fourier spectra along the *k*_*x*_ and *k*_*y*_ momenta, respectively (indicated by the two white dashed lines in Fig. 3d). As can be seen, the measured SSs connect the projections of the two bulk nodal points (indicated by the blue dots) from the same nodal ring, matching well with the simulated SS dispersion (indicated by the red dots).

### ***PT***-related hinge states

**Fig. 4:**
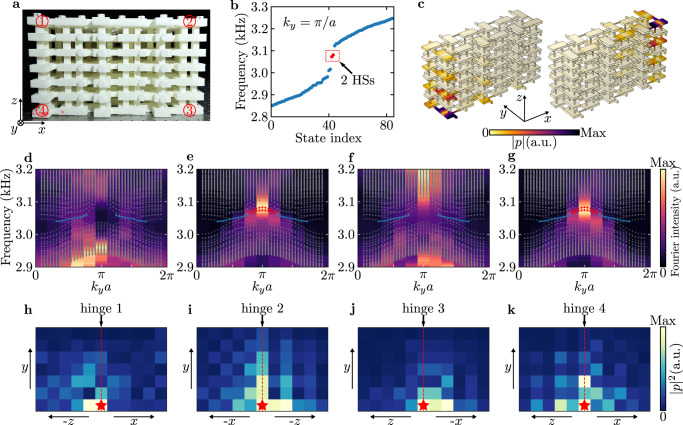
Observation of ***PT***-related hinge states. **a** Experimental sample for measuring the hinge states (HSs). The numbers—label the four *y*-directional hinges. **b** Eigen frequencies for the sample shown in **a** at *k*_*y*_ = *π*/*a*, obtained from full-wave simulation. The blue dots represent the bulk and surface states, and the red dots indicate the HSs. **c**, Eigen profiles for the two states highlighted in red in **b** (∣*p*∣ denotes the absolute value of acoustic pressure). The color indicates the amplitude of the acoustic pressure. **d**–**g** Experimentally measured dispersions for hinge 1 **d**, hinge 2 **e**, hinge 3 **f**, and hinge 4 **g**. The gray dots represent simulated eigenfrequencies of the bulk and SSs, and the red dots indicate simulated hinge bands. The solid blue lines denote the projections of the nodal rings. **h**–**k** Experimentally measured acoustic intensity distributions on two surfaces adjacent to hinge 1 **h**, hinge 2 **i**, hinge 3 **j**, and hinge 4 **k**. The red star indicates the position of the speaker and the red dashed line highlights the position of the hinge. The operating frequencies of the speaker are chosen as: 3078 Hz (hinge 1 and hinge 3), 3076 Hz (hinge 2) and 3080 Hz (hinge 4), which are around the eigenfrequencies of the HSs.

Next, we study the unconventional higher-order topology in this acoustic crystal induced by the nontrivial second SW number *w*_2_. Let us first consider a sample with a simple rectangular geometry as shown in Fig. 4a. This sample consists of six (seven) cavities in the *x* (*z*) direction, thus preserving the required *PT* symmetry. By imposing periodic boundary condition along the *y* direction, we can numerically compute the dispersion for *y*-directional hinges. The results for *k*_*y*_ = *π*/*a* are shown in Fig. 4b and the results for all *k*_*y*_ are given as colored dots in Fig. 4d–g. As can be seen, there are two bands connecting the projections of the nodal rings. The eigen profiles of these two states are given in Fig. 4c, showing they are indeed the hinge Fermi arcs states. Notably, the HSs only exist on two of the four hinges related by the *PT* symmetry. Interestingly, the locations of the HSs can be transferred from the two off-diagonal hinges to the two diagonal ones by simply reversing the dimerization along *z* (i.e., swap the values of *d*_*z*1_ and *d*_*z*2_; see Supplementary Fig. S10).

To probe the hinge Fermi arcs, we place a speaker at the hinge and scan the acoustic field along the hinge. This experiment is repeated for all four hinges (see the labelings of the hinges in Fig. 4a) and the measured dispersions are plotted in Fig. 4d–g. For hinge 2 (Fig. 4e) and hinge 4 (Fig. 4g), the measured dispersions match with the simulated hinge Fermi arcs (red dots), suggesting the existence of HSs on these two hinges. In contrast, the excited states are bulk states when the speaker is placed at hinge 1 (Fig. 4d) or hinge 3 (Fig. 4f). These results demonstrate the off-diagonal distribution (i.e., only at hinge 2 and hinge 4) of the HSs. In addition to the momentum space results, we also conduct real space measurements to reveal the HSs. For each hinge, a speaker operating at the HS’s frequency is placed at one end and the acoustic field on the two adjacent surfaces is measured. As shown in Fig. 4h–k, the measured acoustic intensity distributions exhibit a hinge localization profile only for hinge 2 (Fig. 4i) and hinge 4 (Fig. 4k), which is consistent with the information from the simulated and measured dispersions. Note that here the HSs do not show a clear propagation along the hinge due to their small group velocity and the system’s loss. Nevertheless, the strong field enhancement along the hinge is a clear signature of the HSs. We also note that the system’s loss, which can be modeled as a uniform imaginary number in the diagonal terms of the Hamiltonian, will not change the band topology.

**Fig. 5:**
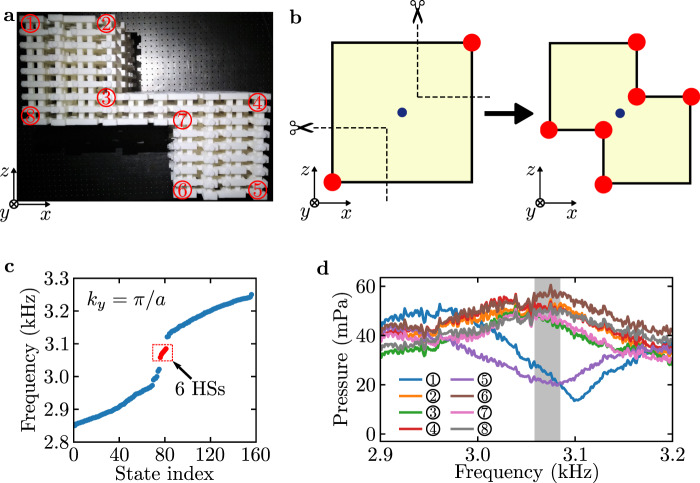
Increased number of hinge states. **a** Experimental sample for demonstrating the odd number of pairs of *PT*-related hinge states (HSs). The numbers—label the eight *y*-directional hinges. **b** Illustration of the process of obtaining an irregularly shaped sample supporting three pairs of HSs by cutting two corners of a rectangle sample with one pair of HSs. Here the red circles represent the HSs and the blue circles denote the inversion centers of the samples. **c** Eigen frequencies for the sample shown in **a** at *k*_*y*_ = *π*/*a*, obtained from full-wave simulation. The blue dots represent the bulk and SSs, and the red dots indicate the HSs. **d** Experimentally measured transmission spectra at the eight hinges. The gray region indicates the frequency range of the HSs.

A fascinating aspect of this acoustic crystal is that the protecting symmetry, i.e., the *PT* symmetry, can be easily preserved in various geometries, not limited to regular ones like the square and rectangular geometries. Furthermore, under different geometries with the same bulk invariant *w*_2_, the forms of the HSs (e.g., the locations and number of HSs) can also be different (see Supplementary section 5 and Supplementary Fig. S8). This allows us to steer the HSs even without changing the system parameters. To demonstrate this property, we construct a sample with an irregular shape in the *xz* plane, as shown in Fig. 5a. One can imagine a cutting procedure illustrated in Fig. 5b, where the two off-diagonal hinges of a rectangle-shaped sample are cut to get this irregularly-shaped sample. During such a process, the *PT* symmetry remains intact while the pair of hinges that support the HSs are removed. Interestingly, the generated new sample host three, instead of one, pair of *PT*-related HSs (see Fig. 5c). To demonstrate this phenomenon, we measure the transmission spectra at the eight hinges of this sample. As shown in Fig. 5d, in the frequency range of the HSs, the signals measured at hinge 1 and hinge 5 are much lower than the signals measured at other six hinges. This indicates there are no HSs at hinge 1 and hinge 5, consistent with the simulation (see Supplementary Fig. S11). The existence/absence of the HS at each hinge is also confirmed by real-sapce field measurements, as given in Supplementary Fig. S13.

## Discussion

In summary, we have demonstrated an acoustic real nodal-line crystal in the nontrivial SW class, hosting ordinary drumhead SSs and unconventional *PT*-related HSs featuring exotic properties. Our study opens a new route to experimental studies on band topology constructed from real fiber bundles that were hardly explored previously. While our demonstration is in acoustics, the proposed idea can also be generalized to other classical wave systems with *PT* symmetry, including photonic, mechanical and circuit systems^41–44^. Other intriguing properties such as the fractional charges and filling anomalies can also be demonstrated with more specialized measurements^45^. Besides, our results reveal the power of projective symmetries in realizing novel topological phases of matter, which could inspire more topological designs using artificial structures with gauge flux. On the practical level, the *PT*-related HSs, with superior tunability in their number and configuration compared to previous higher-order topological states, can provide robust and reconfigurable control over sound and other classical waves.

## Methods

### Concrete form of the model and its symmetry operators

Since each unit cell consists of 2 × 2 × 2 sites, we assign three sets of the standard Pauli matrices *σ*, *τ* and *ρ* for the three dimensions *x*, *y,* and *z*, respectively. Moreover, we introduce the seven Hermitian 8 × 8 Dirac matrices as

$$\begin{array}{ll}&{{{\Gamma }}}_{1}={\rho }_{3}\otimes {\tau }_{3}\otimes {\sigma }_{2},\quad {{{\Gamma }}}_{2}={\rho }_{3}\otimes {\tau }_{3}\otimes {\sigma }_{1},\\ &{{{\Gamma }}}_{3}={\rho }_{3}\otimes {\tau }_{2}\otimes {\sigma }_{0},\quad {{{\Gamma }}}_{4}={\rho }_{3}\otimes {\tau }_{1}\otimes {\sigma }_{0},\\ &{{{\Gamma }}}_{5}={\rho }_{2}\otimes {\tau }_{0}\otimes {\sigma }_{0},\quad {{{\Gamma }}}_{6}={\rho }_{1}\otimes {\tau }_{0}\otimes {\sigma }_{0},\\ &{{{\Gamma }}}_{7}={\rho }_{3}\otimes {\tau }_{3}\otimes {\sigma }_{3}.\hfill\end{array}$$

The Dirac matrices Γ^*α*^ with *α* = 1, 2 ⋯  , 7 satisfy {Γ^*α*^, Γ^*β*^} = 2*δ*^*αβ*^. With the Dirac matrices, the tight-binding Hamiltonian in momentum space is given by

3 $$H({{{{{{{\bf{k}}}}}}}})=	 \mathop{\sum}\limits_{i,a}{f}_{i,a}({k}_{i}){{{\Gamma }}}_{2i-a+1} \\ 	+{g}_{x,1}({k}_{x}){{{{{{{\rm{i}}}}}}}}{{{\Gamma }}}_{2}{{{\Gamma }}}_{3}{{{\Gamma }}}_{4}+{g}_{x,2}({k}_{x}){{{{{{{\rm{i}}}}}}}}{{{\Gamma }}}_{1}{{{\Gamma }}}_{3}{{{\Gamma }}}_{4}\\ 	+{g}_{z,1}({k}_{z}){{{{{{{\rm{i}}}}}}}}{{{\Gamma }}}_{5}{{{\Gamma }}}_{7}+{g}_{z,2}({k}_{z}){{{{{{{\rm{i}}}}}}}}{{{\Gamma }}}_{6}{{{\Gamma }}}_{7}$$

Let *t* denote the hopping magnitude along the undimerized *y* direction, and ${J}_{1}^{x,z}$ and ${J}_{2}^{x,z}$ be the two hopping magnitudes along the two dimerized directions *x* and *z*, respectively. Then, the dimerization strengths are measured by ${J}_{-}^{x,z}/{J}_{+}^{x,z}$ with ${J}_{\pm }^{x,z}=({J}_{1}^{x,z}\pm {J}_{2}^{x,z})/2$. The coefficient functions are given by ${f}_{x,1}({k}_{x})=-{J}_{+}^{x}(1+\cos {k}_{x})$, ${f}_{x,2}={J}_{+}^{x}\sin {k}_{x}$, ${f}_{y,1}({k}_{y})=-t(1+\cos {k}_{y})$, ${f}_{y,2}({k}_{y})=t\sin {k}_{y}$, ${f}_{z,1}({k}_{z})={J}_{+}^{z}(1+\cos {k}_{z})$, ${f}_{z,2}({k}_{z})=-{J}_{+}^{z}\sin {k}_{z}$, and ${g}_{x,1}({k}_{x})=-{J}_{-}^{x}(1-\cos {k}_{x})$, ${g}_{x,2}({k}_{x})=-{J}_{-}^{x}\sin {k}_{x}$, ${g}_{z,1}({k}_{z})=-{J}_{-}^{z}(1-\cos {k}_{z})$, ${g}_{z,2}({k}_{z})={J}_{-}^{z}\sin {k}_{z}$.

The momentum-space symmetry operators for M_*i*_ are given by

$$\begin{array}{ll}&{{{{{{{{\mathcal{M}}}}}}}}}_{x}={\rho }_{0}\otimes {\tau }_{0}\otimes {\sigma }_{1}\,{\hat{m}}_{x}=-{{{{{{{\rm{i}}}}}}}}{{{\Gamma }}}_{1}{{{\Gamma }}}_{7}{\hat{m}}_{x},\\ &{{{{{{{{\mathcal{M}}}}}}}}}_{y}={\rho }_{0}\otimes {\tau }_{1}\otimes {\sigma }_{3}\,{\hat{m}}_{y}=-{{{{{{{\rm{i}}}}}}}}{{{\Gamma }}}_{3}{{{\Gamma }}}_{7}{\hat{m}}_{y},\\ &{{{{{{{{\mathcal{M}}}}}}}}}_{z}={\rho }_{1}\otimes {\tau }_{3}\otimes {\sigma }_{3}\,{\hat{m}}_{z}=-{{{{{{{\rm{i}}}}}}}}{{{\Gamma }}}_{5}{{{\Gamma }}}_{7}{\hat{m}}_{z},\\ \end{array}$$

where each ${\hat{m}}_{i}$ is the operator sending *k*_*i*_ to − *k*_*i*_ with *i* = *x*, *y*, *z*. It is easy to check the desired projective algebraic relations: $\{{{{{{{{{\mathcal{M}}}}}}}}}_{i},{{{{{{{{\mathcal{M}}}}}}}}}_{j}\}=2{\delta }_{ij}$. The momentum-space operators for the translations L_*i*_ are given by

$$\begin{array}{ll}&{{{{{{{{\mathcal{L}}}}}}}}}_{x}={\rho }_{0}\otimes {\tau }_{0}\otimes \left[\begin{array}{cc}0&{e}^{{{{{{{{\rm{i}}}}}}}}{k}_{x}}\\ 1&0\end{array}\right],\\ &{{{{{{{{\mathcal{L}}}}}}}}}_{y}={\rho }_{0}\otimes \left[\begin{array}{cc}0&{e}^{{{{{{{{\rm{i}}}}}}}}{k}_{y}}\\ 1&0\end{array}\right]\otimes {\sigma }_{3},\\ &{{{{{{{{\mathcal{L}}}}}}}}}_{z}=\left[\begin{array}{cc}0&{e}^{{{{{{{{\rm{i}}}}}}}}{k}_{z}}\\ 1&0\end{array}\right]\otimes {\tau }_{3}\otimes {\sigma }_{3}.\end{array}$$

We see that ${{{{{{{{\mathcal{L}}}}}}}}}_{i}^{2}={e}^{{{{{{{{\rm{i}}}}}}}}{k}_{i}}$ for *i* = *x*, *y*, *z*, and $\{{{{{{{{{\mathcal{L}}}}}}}}}_{i},{{{{{{{{\mathcal{L}}}}}}}}}_{j}\}=0$ if *i* ≠ *j*. One can furthermore to check the projective algebraic relations between ${{{{{{{{\mathcal{L}}}}}}}}}_{i}$ and ${{{{{{{{\mathcal{M}}}}}}}}}_{j}$: $\{{{{{{{{{\mathcal{M}}}}}}}}}_{i},{{{{{{{{\mathcal{L}}}}}}}}}_{j}\}=0$ if *i* ≠ *j*, and ${{{{{{{{\mathcal{M}}}}}}}}}_{i}{{{{{{{{\mathcal{L}}}}}}}}}_{i}{{{{{{{{\mathcal{M}}}}}}}}}_{i}=-{{{{{{{{\mathcal{L}}}}}}}}}_{i}^{{{{\dagger}}} }$ for *i*, *j* = *x*, *y*, *z*.

It is easy to check that all symmetry operators ${{{{{{{{\mathcal{L}}}}}}}}}_{i}$ and ${{{{{{{{\mathcal{M}}}}}}}}}_{j}$ commute with time-reversal operator ${{{{{{{\mathcal{T}}}}}}}}={{{{{{{\mathcal{K}}}}}}}}\hat{I}$ with $\hat{I}$ the inversion of **k** and ${{{{{{{\mathcal{K}}}}}}}}$ the complex conjugation. Specifically at *K* = (*π*, *π*, *π*), we see

$${{{{{{{{\mathcal{L}}}}}}}}}_{x}^{K}={\rho }_{0}\otimes {\tau }_{0}\otimes (-{{{{{{{\rm{i}}}}}}}}{\sigma }_{2}),\\ {{{{{{{{\mathcal{L}}}}}}}}}_{y}^{K}={\rho }_{0}\otimes (-{{{{{{{\rm{i}}}}}}}}{\tau }_{2})\otimes {\sigma }_{3},\\ {{{{{{{{\mathcal{L}}}}}}}}}_{z}^{K}=(-{{{{{{{\rm{i}}}}}}}}{\rho }_{2})\otimes {\tau }_{3}\otimes {\sigma }_{3}.$$

Together with operators ${{{{{{{{\mathcal{M}}}}}}}}}_{i}$ above, we can verify that the projective algebraic relations (2) at *K* are indeed satisfied.

In the absence of dimerization, i.e., ${J}_{-}^{x}={J}_{-}^{z}=0$, it is straightforward to check that all ${{{{{{{{\mathcal{M}}}}}}}}}_{i}$ and ${{{{{{{{\mathcal{L}}}}}}}}}_{j}$ commute with the Hamiltonian (3). After the dimerization patterns ${{\mathfrak{D}}}_{1}$ and ${{\mathfrak{D}}}_{2}$ are introduced, all L_*i*_ and M_*j*_ are broken, and therefore ${{{{{{{{\mathcal{M}}}}}}}}}_{i}$ and ${{{{{{{{\mathcal{L}}}}}}}}}_{j}$ do not commute with (3) any more. Nevertheless, the off-centered inversion symmetry P = L_*y*_L_*z*_M_*x*_M_*y*_M_*z*_ is preserved, and the momentum-space operator ${{{{{{{\mathcal{P}}}}}}}}$ for P can be derived as a product of the corresponding operators presented above. Then, the P*T* symmetry operator ${{{{{{{\mathcal{P}}}}}}}}{{{{{{{\mathcal{T}}}}}}}}$ is given by

4 $$\begin{array}{rcl}&&{{{{{{{\mathcal{P}}}}}}}}{{{{{{{\mathcal{T}}}}}}}}=\left[\begin{array}{cc}{e}^{{{{{{{{\rm{i}}}}}}}}{k}_{z}}&0\\ 0&1\end{array}\right]\otimes \left[\begin{array}{cc}-{e}^{{{{{{{{\rm{i}}}}}}}}{k}_{y}}&0\\ 0&-1\end{array}\right]\otimes {\sigma }_{1}{{{{{{{\mathcal{K}}}}}}}}.\end{array}$$

It is straightforward to check ${{{{{{{\mathcal{P}}}}}}}}{{{{{{{\mathcal{T}}}}}}}}$ commutes with the Hamiltonian (3) even with nonzero ${J}_{-}^{x}$ and ${J}_{-}^{z}$.

With small dimerizations ${{\mathfrak{D}}}_{1}$ and ${{\mathfrak{D}}}_{2}$, the low-energy effective model can be derived in the vicinity of each nodal line. Each low-energy effective model can be cast into the form of (1), namely the real Dirac model with a “partial mass term” along the *z* direction, by appropriately choosing the basis of four low-energy modes. The 4 × 4 Hermitian Dirac matrices in (1) can be chosen as

$$\begin{array}{ll}{\gamma }_{1}={\sigma }_{1}\otimes {\tau }_{0},\quad {\gamma }_{2}={\sigma }_{2}\otimes {\tau }_{2},\quad {\gamma }_{3}={\sigma }_{3}\otimes {\tau }_{0}\\ \quad{\gamma }_{4}={\sigma }_{2}\otimes {\tau }_{3},\quad{\gamma }_{5}={\sigma }_{2}\otimes {\tau }_{1}.\end{array}$$

Here, *σ* and *τ* are two sets of the standard Pauli matrices, which have no direct relation with those used to define Γ^*α*^.

### Full-wave simulation

All numerical simulations of the acoustic model are performed in the commercial software Comsol Multiphysics, pressure acoustics module. The software solves the Helmholtz equation with the finite element method. In the simulations, periodic boundary conditions with Bloch phase shifts are assigned to the periodic boundaries, while other boundaries are set as sound rigid boundaries due to the large impedance mismatch between the printing materials and air. The sound speed and density of air are set to be 347.2 m/s and 1.16 kg/m^3^, respectively. The geometrical parameters of the acoustic model are given in the caption of Fig. 3.

To get the equi-frequency contour of the bulk bands (Fig. 3c), we compute all the eight bulk bands in area 0.5*π*/*a* < *k*_*x*_ < 1.5*π*/*a*, 0 < *k*_*y*_ < *π*/*a* and 0.5*π*/*a*_*z*_ < *k*_*z*_ < 1.5*π*/*a*_*z*_, with 30 computing points in each momentum direction. The contour at the other side of the Brillouin zone is obtained through the time-reversal operation. In the simulation of surface dispersion (Fig. 3d–f), we adopt an acoustic supercell with periodic boundary condition along the *x* and *y* directions and 21 cavities along the *z* direction. In the simulations of the hinge dispersions (Figs. 4b–g and 5c), the simulated geometries in the *xz* plane are the same as the experimental samples (see Figs. 4a and 5a), with periodic boundary conditions imposed for the *y* direction.

### Sample design and fabrication

To implement the sound rigid walls that surround the air cavities and tubes, we design hard walls with a thickness of 5 mm to cover the whole sample. These walls are thick enough to provide the rigid wall condition. In order to excite and measure the sound signals, we drill two small holes (with radii of 5 mm) on the boundary cavities. These holes are covered with size-matched plugs when they are not in use.

The samples are fabricated through the stereolithography apparatus technique, with a fabrication resolution of around 0.1 mm. The dimensions of the three samples (i.e., the samples shown in Figs. 3a, 4a and 5a) are around 1.5 m × 1.5 m × 0.2 m, 0.4 m × 1.5 m × 0.2 m and 0.7 m × 0.5 m × 0.5 m, respectively. Due to their large sizes, these samples are fabricated as separate parts and then assembled together.

### Experimental measurement

All experiments are conducted using the same scheme, as illustrated in Fig. S9 in Supplementary Information. The sound signal is launched by a speaker (Tymphany PMT-40N25AL01-04) placed on the surface (for measuring the SSs) or the hinge (for measuring the HSs). The speaker generates a broadband sound signal from 2000 Hz to 4000 Hz, which covers the frequency range of our interested bands. Two microphones (Brüel & Kjær Type 4182) are used to detect the amplitude and phase of sound in the sample. One of the microphones is placed at the position of the source, serving as a reference probe. To ensure the accuracy of the frequency-resolved spectra, we have also checked that there are no resonances in the spectrum of the source (see Supplementary Fig. S12). The other microphone scan field distributions in the targeted areas in the sample. The measured signal is processed by an analyzer (Brüel & Kjær 3160-A-022 module) to obtain the experimental data with the amplitude and phase of sound at each measured point for the frequencies ranging from 0 Hz to 6400 Hz (the frequency resolution is 1 Hz).

## Supplementary information

Supplementary Information

Peer Review File

## Data Availability

The experimental data are available in the data repository for Nanyang Technological University at 10.21979/N9/NEB0G4. Other data that support the findings of this study are available from the corresponding authors upon reasonable request.
