# Supplementary material for: Stiefel-Whitney topological charges in a three-dimensional acoustic nodal-line crystal
Source: Nat Commun. 2023 Jul 28;14:4563. doi: 10.1038/s41467-023-40252-7 (PMC10382567; doi:10.1038/s41467-023-40252-7)
Supplement: Supplementary file 1 — Supplementary Information [file 41467_2023_40252_MOESM1_ESM.pdf]

Supplemental Information for "Stiefel-Whitney topological charges in a three-dimensional acoustic nodal-line crystal"

Haoran Xue,[1] Z. Y. Chen,[2] Zheyu Cheng,[1] J. X. Dai,[2]
Yang Long,[1] Y. X. Zhao,[3,4,*] and Baile Zhang[1,5,†]

[1]*Division of Physics and Applied Physics,
School of Physical and Mathematical Sciences,
Nanyang Technological University, Singapore 637371, Singapore*

[2]*National Laboratory of Solid State Microstructures and Department of Physics,
Nanjing University, Nanjing 210093, China*

[3]*Department of Physics and HKU-UCAS Joint Institute
for Theoretical and Computational Physics at Hong Kong,
The University of Hong Kong, Pokfulam Road, Hong Kong, China*

[4]*HK Institute of Quantum Science & Technology,
The University of Hong Kong, Pokfulam Road, Hong Kong, China*

[5]*Centre for Disruptive Photonic Technologies,
Nanyang Technological University, Singapore 637371, Singapore*

1. THE FIRST AND SECOND STIEFEL-WHITNEY NUMBERS

1.1 The classifying space of spacetime-inversion-invariant systems

For spacetime-inversion-symmetric (PT-symmetric) systems with $(\mathcal{PT})^2 = 1$, the PT symmetry operator can always be represented in an appropriate basis as $\hat{\mathcal{P}}\hat{\mathcal{T}} = \hat{\mathcal{K}}$, with $\hat{\mathcal{K}}$ the complex conjugation. In momentum space, the commutation relation between $\hat{\mathcal{P}}\hat{\mathcal{T}}$ and the Hamiltonian $\mathcal{H}(\mathbf{k})$ requires $\mathcal{H}(\mathbf{k})$ to be real [1]. Here, let us introduce the flattened Hamiltonian $\widetilde{\mathcal{H}} = \mathrm{sgn}(\mathcal{H})$, which is obtained by continuously deforming the energies of conduction/valence bands of $\mathcal{H}(\mathbf{k})$ into ± 1. $\widetilde{\mathcal{H}}(\mathbf{k})$ has the same topological structure as $\mathcal{H}(\mathbf{k})$, and can be diagonalized as:

$$\widetilde{\mathcal{H}}(\mathbf{k}) = \mathcal{O}(\mathbf{k}) \begin{bmatrix} I_M & \\ & -I_N \end{bmatrix} \mathcal{O}^T(\mathbf{k}), \tag{S1}$$

where $\mathcal{O} \in \mathrm{O}(M+N)$ is a real orthogonal matrix and $I_{M/N}$ denotes the M/N-dimensional identity matrix. $\widetilde{\mathcal{H}}(\mathbf{k})$ is clearly invariant under the gauge transformation:

$$\mathcal{O}(\mathbf{k}) \mapsto \mathcal{O}(\mathbf{k}) \begin{bmatrix} \mathcal{O}_1(\mathbf{k}) & 0 \\ 0 & \mathcal{O}_2(\mathbf{k}) \end{bmatrix}, \tag{S2}$$

with $\mathcal{O}_1 \in \mathrm{O}(M)$ and $\mathcal{O}_2 \in \mathrm{O}(N)$. Therefore, the classifying space of gapped PT-symmetric systems is given by

$$R = \frac{\mathrm{O}(M+N)}{\mathrm{O}(M) \times \mathrm{O}(N)}. \tag{S3}$$

Since $\pi_1(R) \cong \mathbb{Z}_2$ and $\pi_2(R) \cong \mathbb{Z}_2$, gapped PT-symmetric systems have $\mathbb{Z}_2$ classification in both 1D and 2D. Corresponding topological invariants are just the first Stiefel-Whitney (SW) number w_1 and the second SW number w_2 [2].

1.2 Definition and calculation methods of w_1 and w_2

w_1 is defined in 1D. As shown in Fig. S1a, the base space is a circle, which is divided into north and south hemicircle $D^1_{N/S}$ with the intersection $S^0 = \{p_0, p_1\}$. Real valence eigenfunctions $|-, m\rangle$ satisfying $|-, m\rangle = |-, m\rangle^*$ defined on D^1_N and D^1_S are glued by the transition function $t(\phi) \in \mathrm{O}(N)$ on the equator S^0, i.e.,

$$|-, m\rangle_N|_{S^0} = \sum_{n=1}^{N} t_{mn}(\phi)|-, n\rangle_S|_{S^0}. \tag{S4}$$

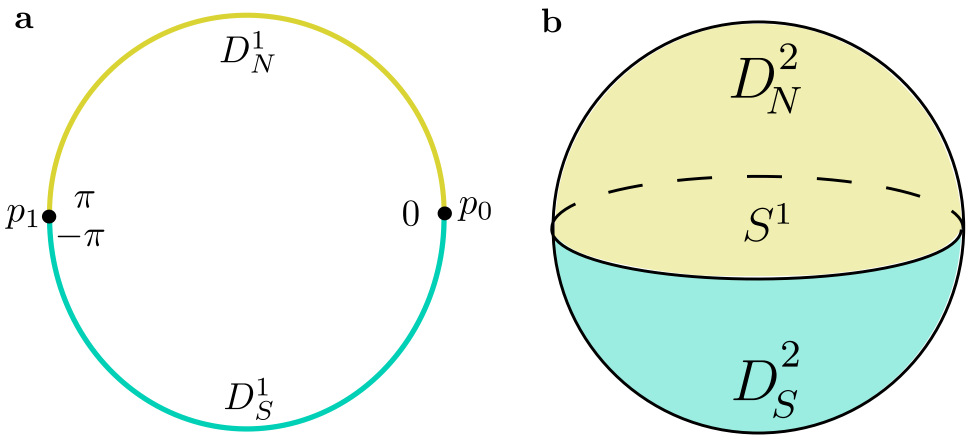

Fig. S1. **Deifinition of the topological invariants.** **a**, A circle S^1 is divided into two semicircles D_N^1 and D_S^1 overlapping at the equator $S^0 = \{p_0, p_1\}$. **b**, The assuming base space in 2D is a sphere S^2, which is divided into two hemispheres D_N^2 and D_S^2 overlapping on the equator S^1.

Then $\pi_0[\mathrm{O}(N)] \cong \mathbb{Z}_2$ imposes obstructions to the globally well-defined basis of real valence eigenfunctions. The topological invariant w_1 just originates from these obstructions, thus we can calculate w_1 by

$$(-1)^{w_1} = \det[t(p_0)]\det[t(p_1)]. \tag{S5}$$

As for w_2, we consider the 2D sphere S^2 in Fig. S1b, which is divided into north and south hemisphere $D_{N/S}^2$ with the intersection S^1. Real valence eigenfunctions $|-, m\rangle$ defined on D_N^2 and D_S^2 are glued by the transition function $t(\phi) \in \mathrm{O}(N)$ on the equator S^1, i.e.,

$$|-, m\rangle_N|_{S^1} = \sum_{n=1}^{N} t_{mn}(\phi)|-, n\rangle_S|_{S^1}. \tag{S6}$$

Then $\pi_1[\mathrm{O}(N)] \cong \mathbb{Z}_2$ imposes obstructions to the globally well-defined basis of real valence eigenfunctions. The topological invariant w_2 just originates from these obstructions. Hence, the topological information is encoded in the phase factors $\theta(\phi)$ of the N eigenvalues of $t(\phi)$.

Now, we introduce the Wilson loop method as

$$W(k_y) = \mathscr{P}\exp\left(-i\int_{C_{k_y}} dk_x\ \mathcal{A}(\mathbf{k})\right). \tag{S7}$$

where $\mathscr{P}$ indicates the path order along circles C_{k_y} (k_y is fixed for each circle) parameterized by k_x. And the non-abelian Berry connection $\mathcal{A}(\mathbf{k})$ is obtained by the valence eigenfunctions as $\mathcal{A}_{\alpha\beta}(\mathbf{k}) = \langle -, \alpha|\partial_{k_x}|-, \beta\rangle$. Here, the gauge of valence functions $|-\alpha\rangle$ has no effect on the eigenvalues of $W(k_y)$. Actually, $W(k_y)$ has the same eigenvalues as the transition function

of the real valence eigenfunctions under the parallel transport gauge. Considering $\theta(k_y)$ as the phase factors of the eigenvalues of $W(k_y)$, the topological invariant w_2 can be derived as

$$w_2 = \xi \mod 2, \tag{S8}$$

where ξ is the times that the trajectories of $\theta(k_y)$ cross $\theta = \pi$

Moreover, w_2 can also be calculated by the real Berry curvature $\mathcal{F}_R$, which is derived from the real Berry connection $\mathcal{A}^R_{mn}(\mathbf{k}) = \langle -, m|d|-, n\rangle$. If $N = 2$, w_2 is just given by

$$w_2 = \frac{1}{4\pi}\int_{S^2} \mathrm{tr}(i\sigma_2\mathcal{F}_R) \mod 2, \tag{S9}$$

where $i\sigma_2$ is the generator of SO(2) rotation in the 2D Euclidean space spanned by the real valence eigenstates with σ_2 the second Pauli matrix.

2. DIRAC MODELS

2.1 The 2D Stiefel-Whitney insulator represented by the Dirac model

We construct a Dirac model for the PT-symmetric insulator in 2D. To preserve $\hat{\mathcal{P}}\hat{\mathcal{T}} = \hat{\mathcal{K}}$, we need three real Dirac matrices, of which two are for the kinetic terms and one is for the mass term. Therefore, the minimal dimension for Dirac matrices is four, and the corresponding Dirac Hamiltonian in momentum space is given by

$$\mathcal{H}_0(\mathbf{k}) = \sin k_x\gamma^1 + \sin k_y\gamma^2 + (M - \cos k_x - \cos k_y)\gamma^3, \tag{S10}$$

where 4×4 Hermitian Dirac matrices are given by

$$\gamma^1 = \sigma_0\otimes\tau_1,\ \gamma^2 = \sigma_0\otimes\tau_3,\ \gamma^3 = \sigma_2\otimes\tau_2,\ \gamma^4 = \sigma_1\otimes\tau_2,\ \gamma^5 = \sigma_3\otimes\tau_2. \tag{S11}$$

They satisfy the Clifford algebra $\{\gamma^i, \gamma^j\} = 2\delta^{ij}1_4$ with 1_4 the 4×4 identity matrix. It is observed that $\mathcal{H}_0(\mathbf{k})$ is gapped if $|M| \neq 2$ and $M \neq 0$. As for the topological invariant, we can apply the Eq. (S9) to calculate w_2, and obtain $w_2 = 1$ for $|M| \in (0, 2)$ while $w_2 = 0$ for $|M| > 2$. This is also verified by the Wilson loop method, as shown in Fig. S2.

$\mathcal{H}_0(\mathbf{k})$ is clearly a first-order topological insulator with helical edge states on each edge, which is shown in Fig. S3a. However, let us consider adding to $\mathcal{H}_0(\mathbf{k})$ in Eq. (S10) the following PT-symmetric perturbations

$$\Delta\mathcal{H} = i\gamma^1(m_1\gamma^4 + m_3\gamma^5) + i\gamma^2(m_2\gamma^4 + m_4\gamma^5), \tag{S12}$$

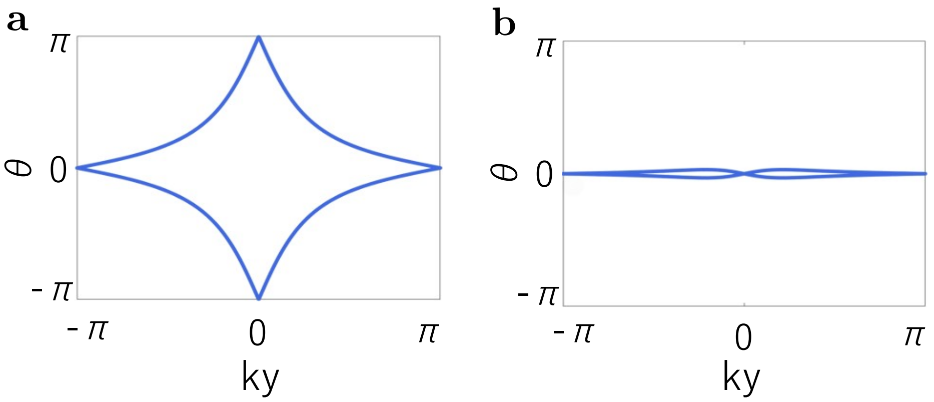

Fig. S2. **Topology of the 2D Dirac model.** The Wilson loop spectrum of $\mathcal{H}_0(\mathbf{k})$ in Eq. (S10) with $M = 1$ for **a** and $M = 3$ for **b**. Clearly, the topological invariants are $w_2 = 1$ for **a** and $w_2 = 0$ for **b**.

which are the only PT-invariant relevant quadratic perturbations. The Hamiltonian

$$\mathcal{H}(\mathbf{k}) = \mathcal{H}_0(\mathbf{k}) + \Delta\mathcal{H} \tag{S13}$$

possesses a nontrivial bulk-boundary correspondence: with unchanged bulk topological invariant w_2, the first-order boundaries undergo transitions separating different phases with second-order boundary zero modes. Although $\Delta\mathcal{H}$ is just perturbations and the bulk gap is not closed, i.e., the bulk topological invariant is unchanged, the boundary states change with different perturbations. As shown in Fig. S3b,c, $\mathcal{H}(\mathbf{k})$ describes the first-order topological phases with a pair of PT-related helical edge states along the x/y direction if $m_{1/2} = m_{3/4} = 0$. Meanwhile, $\mathcal{H}(\mathbf{k})$ corresponds to the second-order topological phase with a pair of PT-related zero-mode corners if

$$(m_1, m_3) = \alpha(m_2, m_4), \;\; \alpha \neq 0. \tag{S14}$$

As shown in the Fig. S3d(e), if $\alpha < 0$ ($\alpha > 0$), there exists a pair of zero-mode states at the diagonal (off-diagonal) corners of the square sample. Therefore, we obtain the phase diagram with respect to m_1 and m_2 in Fig. S3f. It is noteworthy that all the phases share the same topological invariant w_2.

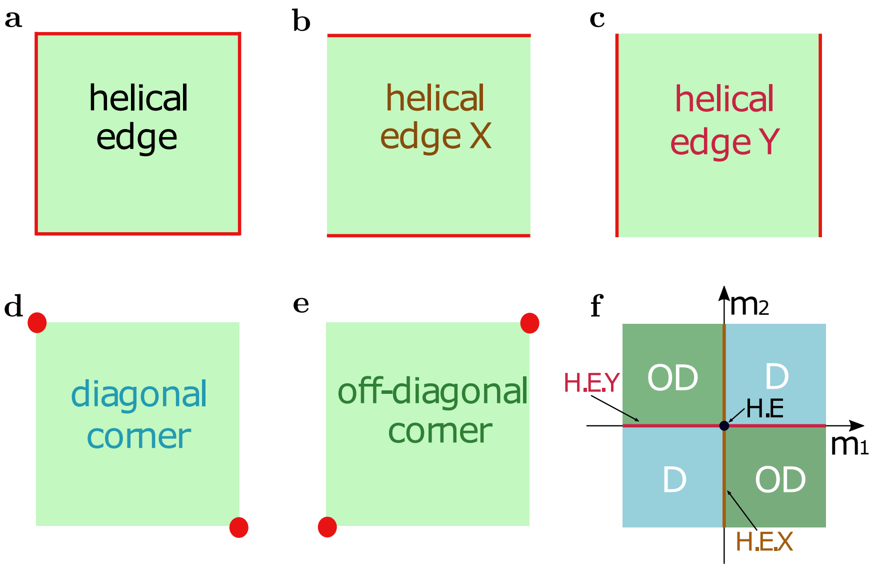

Fig. S3. **Phase diagram of the 2D Dirac model. a-e**, Illustrations of possible topological boundary-mode configurations for $\mathcal{H}(\mathbf{k}) = \mathcal{H}_0(\mathbf{k}) + \Delta\mathcal{H}$. **a**, The critical state [Eq. (S10)] has helical edge modes over all edges. **b** and **c** are states at phase boundaries between **d** and **e**, which has helical edge modes only on a single pair of edges. **d** and **e** illustrate the two second-order topological phases with a single pair of corner zero-modes on diagonal and off-diagonal corners, respectively. **f** shows the phase diagram with respect to m_1 and m_2, where "D", "OD", and "H.E." stand for diagonal, off-diagonal, and helical edge, respectively.

2.2 The *PT*-symmetric 3D semimetal represented by the Dirac model

Based on the Hamiltonian $\mathcal{H}_0(\mathbf{k})$ in Eq. (S10), the PT-symmetric semimetal model in 3D can be constructed as

$$\mathcal{H}_0^{3\mathrm{D}}(\mathbf{k}) = \sin k_x \gamma^1 + \sin k_y \gamma^2 + (M - \cos k_x - \cos k_y - \cos k_z)\gamma^3, \tag{S15}$$

where the Dirac matrices γ^i are given in Eq. (S11). Clearly, $\mathcal{H}_0^{3\mathrm{D}}$ describes a 3D real Dirac semimetal when $1 < M < 3$. Two real Dirac points reside at $(k_x, k_y, k_z) = (0, 0, \pm K_z)$ with $K_z = \arccos(M - 2)$, which is shown in Fig. S4a.

The $k \cdot p$ models for the two real Dirac points are given by

$$\mathcal{H}_0(\mathbf{q}) = q_x \gamma^1 + q_y \gamma^2 \pm 1 v_z q_z \gamma^3, \tag{S16}$$

where $\mathbf{q}$ is measured from each Dirac point, and $v_z = \sin K_z$. Calculating on the 2D sphere $(q_x^2 + q_y^2 + v_z^2 q_z^2) = 1$, we can obtain the topological charge of the Dirac point as $w_2 = 1$ by

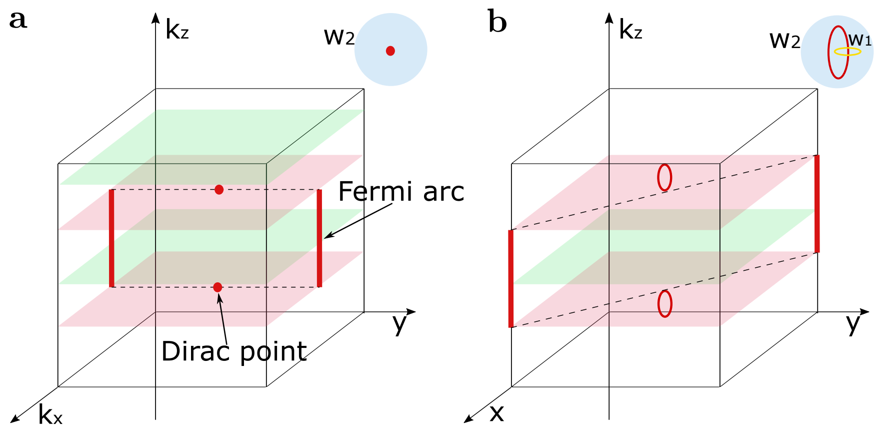

Fig. S4. **The 3D Dirac model.** **a**, For the model $\mathcal{H}_0^{3\mathrm{D}}(\mathbf{k})$ in Eq (S15), there exist Fermi arcs connecting the projections of real Dirac points on the surfaces with breaking the translational symmetry along y-direction. Top-right inset shows that each Dirac point carries a nontrivial w_2 defined on a sphere surrounding it. **b**, Considering the perturbations $\Delta\mathcal{H}$ in Eq. (S13), each Dirac point is shrunk to a nodal loop. And there exist Fermi arcs connecting the projections of real Dirac points on the hinges by breaking the translational symmetry along x-direction and y-direction. Each nodal loop carries a nontrivial w_2 defined on a sphere surrounding it. Meanwhile, there exists a nontrivial first SW number w_1 on a small circle S^1 transversely surrounding the nodal loop.

Eq. (S9). The nontrivial topology leads to the surface Fermi arcs confined by $k_z \in (-K_z, K_z)$ on the side surfaces (Fig. S4a). For two 2D k_x-k_y subsystems on two sides of a chosen Dirac point, respectively, one of them must be topologically nontrivial and the other trivial, since the difference of their w_2's is equal to the nontrivial w_2 of the Dirac point.

Meanwhile, if we add the perturbations $\Delta\mathcal{H}$ in Eq.(S13) to $\mathcal{H}_0^{3\mathrm{D}}(\mathbf{k})$ in Eq. (S15) as

$$\mathcal{H}^{3\mathrm{D}}(\mathbf{k}) = \mathcal{H}_0^{3\mathrm{D}}(\mathbf{k}) + \Delta\mathcal{H}, \tag{S17}$$

the Dirac points are perturbed as $\mathcal{H}^{\pm} = \mathcal{H}_0^{\pm} + \Delta\mathcal{H}$, and one finds that each Dirac point is spread into a nodal loop parallel to the k_z axis (Fig. S4b). Each nodal loop here carries twofold topological charges $(w_1, w_2) = (1, 1)$. First, because they originate from the Dirac points, they inherit the 2D topological charges w_2 of the Dirac points defined on a sphere enclosing each loop. Second, similar to conventional nodal lines, they have the 1D topological charge defined by the first SW number w_1 on a small circle S^1 transversely surrounding them (see the inset of Fig. S4b).

With perturbations $\Delta\mathcal{H}$, the helical edge states of each nontrivial 2D k_x-k_y subsystem are gapped and second-order zero-mode states appear on the corners, thus the surface Fermi arcs generically transform into off-diagonal or diagonal hinge Fermi arcs (Fig. S4b). Similar to before, whether the Fermi arcs distribute on the diagonal or off-diagonal hinges is also determined by the signal of α in Eq. (S14). Corresponding to the phase diagram in Fig. S3f, the phases with off-diagonal and diagonal hinge Fermi arcs are separated by anisotropic critical states with the surface helical Fermi arcs only existing on the $x-z$ or $y-z$ surfaces.

3. TOPOLOGICAL INVARIANTS OF THE LATTICE MODEL

In the main text, the 3D lattice model is given by

$$H(\mathbf{k}) = \sum_{i,a} f_{i,a}(k_i)\Gamma_{2i-a+1} + g_{x,1}(k_x)\mathrm{i}\Gamma_2\Gamma_3\Gamma_4 + g_{x,2}(k_x)\mathrm{i}\Gamma_1\Gamma_3\Gamma_4 + g_{z,1}(k_z)\mathrm{i}\Gamma_5\Gamma_7 + g_{z,2}(k_z)\mathrm{i}\Gamma_6\Gamma_7, \tag{S18}$$

where the coefficient functions are

$$\begin{aligned}
f_{x,1}(k_x) &= -J_+^x(1+\cos k_x), \;\; f_{x,2}(k_x) = J_+^x \sin k_x, \\
f_{y,1}(k_y) &= -t(1+\cos k_y), \qquad f_{y,2}(k_y) = t\sin k_y, \\
f_{z,1}(k_z) &= J_+^z(1+\cos k_z), \qquad f_{z,2}(k_z) = -J_+^z \sin k_z, \\
g_{x,1}(k_x) &= -J_-^x(1-\cos k_x), \;\; g_{x,2}(k_x) = -J_-^x \sin k_x, \\
g_{z,1}(k_z) &= -J_-^z(1-\cos k_z), \;\; g_{z,2}(k_x) = J_-^z \sin k_z.
\end{aligned} \tag{S19}$$

It is noteworthy that $J_\pm^{x,z} = J_1^{x,z} \pm J_2^{x,z}$.

With setting the parameters as $t=1, J_1^x=1, J_2^x=2.2, J_1^z=1, J_2^z=1.6$, we obtain the nodal loop semimetal, which is shown in Fig. S5a. On the 2D sphere $(k_x-c_x)^2+(k_y-c_y)^2+(k_z-c_z)^2=(\pi/2)^2$ enclosing one nodal loop $[(c_x,c_y,c_z)=(\pi,3\pi/2,\pi)$ or $(\pi,\pi/2,\pi)]$, we obtain the Wilson loop spectrum as Fig. S5b. This sphere can also be represented by

$$k_x = c_x + r\sin\varphi\cos\psi,\;\; k_y = c_y + r\sin\varphi\sin\psi,\;\; k_z = c_z + r\cos\varphi, \tag{S20}$$

with $r=\pi/2$, $\varphi\in[0,\pi]$ and $\psi\in[0,2\pi]$. We calculate the Wilson loops along circles C_φ (φ is fixed for each circle) parameterized by ψ. Numerical results show that $w_2=1$ on the sphere, i.e., each nodal loop carries a nontrivial w_2 defined on a sphere surrounding it. On a small circle S^1 transversely surrounding the nodal loop, we can obtain nontrivial w_1.

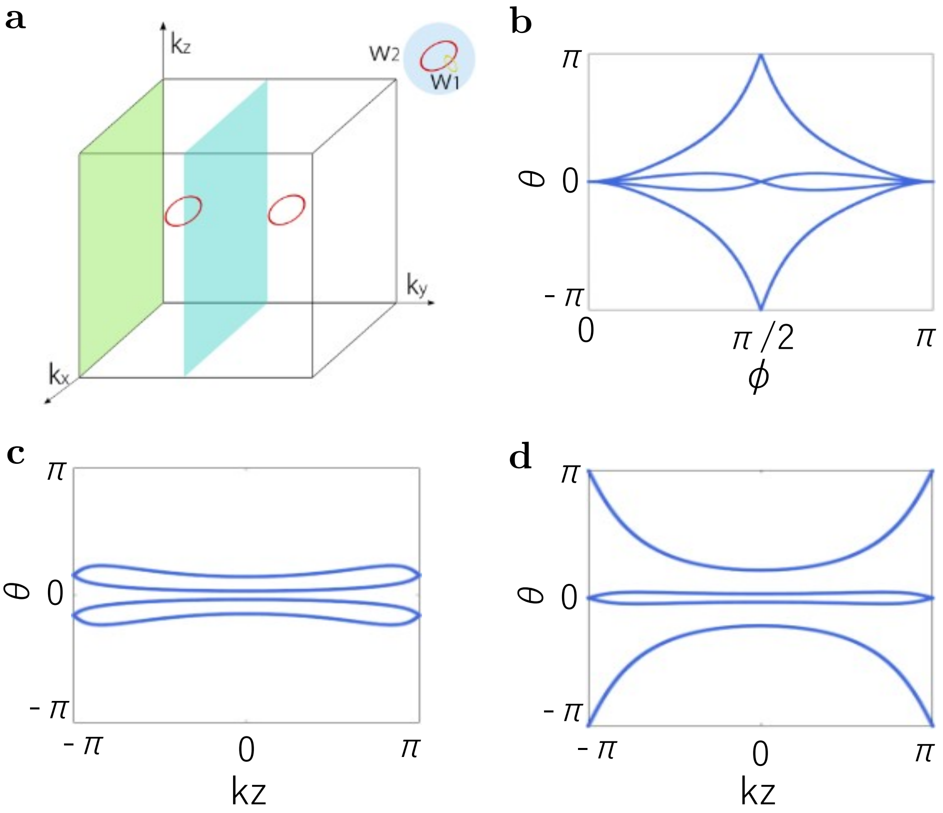

Fig. S5. **Topological invariants of the lattice model.** **a**, The nodal loop semimetal corresponding to the Hamiltonian (S18) with setting the parameters as $t = 1, J_1^x = 1, J_2^x = 2.2, J_1^z = 1, J_2^z = 1.6$. Red rings denote the nodal loops. Green and blue planes are $k_y = 0$ and $k_y = \pi$, respectively. Top-right inset shows that each nodal loop carries a nontrivial w_2 defined on a sphere surrounding it. Meanwhile, there exists nontrivial w_1 on a small circle S^1 transversely surrounding the nodal loop. **b**, The Wilson loop spectrum on the 2D sphere with a radius of $\pi/2$ and its center at point $(c_x, c_y, c_z) = (\pi, \pi/2, \pi)$ (or $(\pi, 3\pi/2, \pi)$), which encloses one nodal loop (encloses the other nodal loop). Here, we use the coordinates ψ and φ as $k_x = c_x + r\sin\varphi\cos\psi,\ k_y = c_y + r\sin\varphi\sin\psi,\ k_z = c_z + r\cos\varphi$. It is observed that w_2 is nontrivial on each sphere. **c** and **d**, The Wilson loop spectra on the green and blue planes in **a**, respectively. Clearly, w_2 is trivial in **c** while nontrivial in **d**.

Similarly, on two sides of a chosen nodal loop, one of them must be topological nontrivial and the other trivial. As shown in Figs. S5c and d, w_2 is trivial and nontrivial on the green plane with $k_y = 0$ and the blue plane with $k_y = \pi$, respectively.

4. DISPERSION OF THE LATTICE MODEL

4.1 Bulk dispersion under coupling dimerizations

In this subsection, we show numerically how the nodal rings are induced from the original eightfold point degeneracy by coupling dimerizations in the x and z directions. Our starting point is the lattice with all couplings being equal. In this case, there should be an eightfold point degeneracy in the Brillouin zone corner (see Fig. S6a; note we shift the Brillouin zone corner to the center of the box for easy visualization), which is enforced by the projective symmetry algebra as discussed in the main text. Our numerical calculation confirms the existence of such a degenerate point (see Fig. S6d). Then, we apply the x-directional coupling dimerization that alternates along the y direction as given in the main text. This splits the eightfold degenerate point into two fourfold real Dirac points along the k_y axis, as shown in Fig. S6b, e. Finally, a z-directional dimerization that alternates along both the x and y directions (see Fig. 1h in the main text) spreads each Dirac point into a nodal ring in the $k_x - k_y$ plane (see Fig. S6c, f). We also plot in Fig. S6g-i the simulated bulk dispersions for the acoustic crystal, which shows a very similar band degeneracy evolution process as we find in the tight-binding model. Besides, we can also see that the acoustic bands match well with the tight-binding ones, which verifies the validity of the acoustic design.

4.2 Surface and hinge dispersions under coupling dimerizations

In this subsection, we show the surface and hinge dispersions under coupling dimerizations in the tight-binding model, as supplements to the simulated and measured dispersions given in the main text. In the absence of dimerizations, there is no boundary state on the surface or at the hinge (see Fig. S7a, d). After applying the x-directional dimerization, there emerge surface states on the yz surface in between the two real Dirac points (see Fig. S7e), while no surface state appears on the xy surface (see Fig. S7b). Finally, with both dimerizations, the surface states on the yz surface are gapped out and mid-gap y-direction hinge states exist (see Fig. S7f). Meanwhile, surface states are also induced on the xy surface (see Fig. S7c). Notably, the surface and hinge states have distinct k_y momenta. The surface states are within the projections of the nodal rings, having the typical drumhead dispersions. The

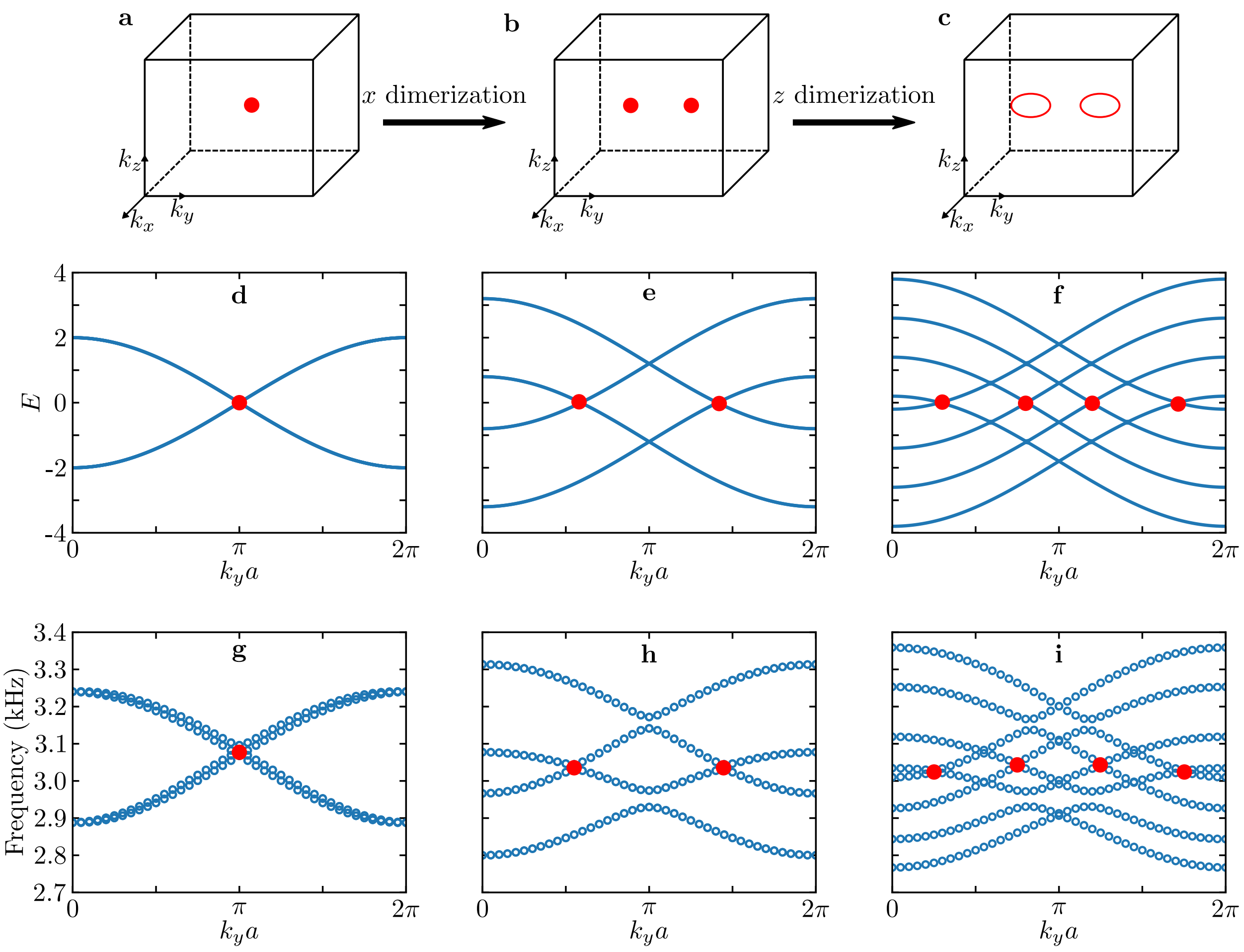

Fig. S6. **Evolution of bulk dispersion under coupling dimerizations. a-c**, Illustration of the evolution of the bulk band degeneracies upon coupling dimerizations. The box denotes the Brillouin zone, with the Brillouin zone corner shifted to the center of the box. The red dots and lines represent the degenerate points at zero energy. **d-f**, Bulk dispersions along k_y ($k_x = k_z = \pi$) calculated using the tight-binding model. The red dots represent the degenerate points at zero energy. The coupling parameters used in the calculations are: $t = J_1^x = J_2^x = J_1^z = J_2^z = 1$ (**d**), $t = 1, J_1^x = 1, J_2^x = 2.2, J_1^z = 1, J_2^z = 1$ (**e**) and $t = 1, J_1^x = 1, J_2^x = 2.2, J_1^z = 1, J_2^z = 1.6$ (**f**). **g-i**, Similar plots to **d-f**, but calculated using the acoustic crystal.

hinge states connect the projections of the nodal rings, forming hinge Fermi arcs.

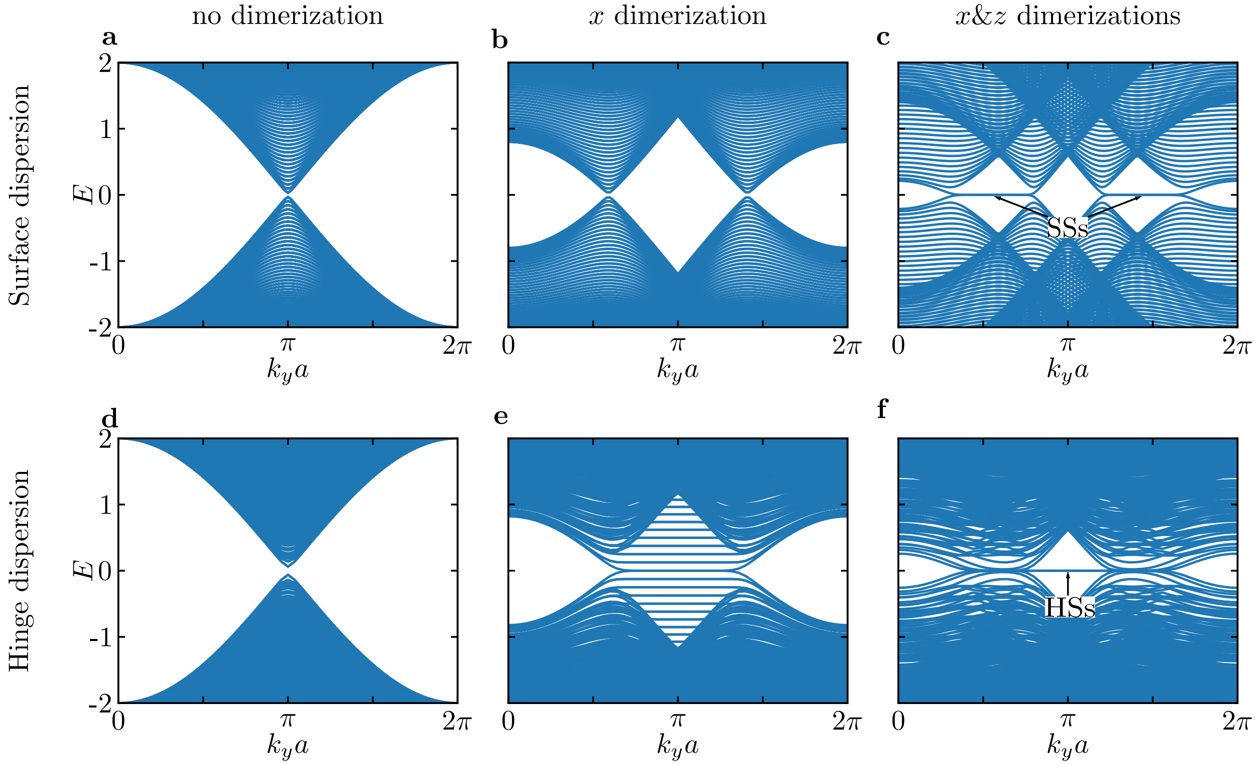

Fig. S7. **Evolution of hinge and surface dispersions under coupling dimerizations. a-c**, Surface dispersions for the no dimerization case (**a**), the x-directional dimerization case (**b**) and the x- and z-directional dimerization case (**c**). The calculations are performed using the tight-binding model under a slab geometry, with periodic boundary condition along the x and y directions and open boundary condition along the z direction. We fix $k_x = \pi$ and use 99 layers in the z direction. **d-f**, Hinge dispersions for the no dimerization case (**d**), the x-directional dimerization case (**e**) and the x- and z-directional dimerization case (**f**). The calculations are performed using the tight-binding model under a tubelike geometry, with periodic boundary condition along the y direction and open boundary condition along the x and z directions. We use 50 and 49 layers in the x and z directions, respectively. The coupling parameters used in the calculations are the same as Fig. 6. HSs: hinge states.

5. *PT*-RELATED HINGE STATES UNDER VARIOUS GEOMETRIES.

In this section, we present more calculations on the distributions of hinge states under several different geometries, as shown in Fig. S8. The calculations are performed using the tight-binding model with tubelike geometries (i.e., with periodic boundary condition along

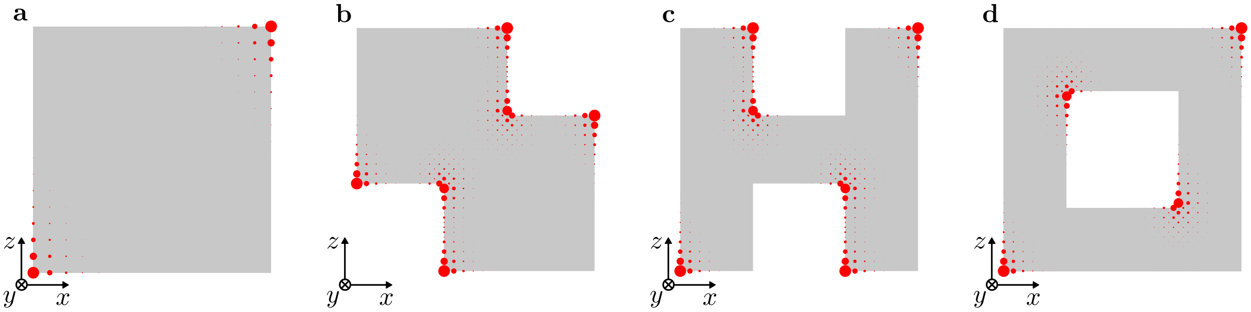

Fig. S8. ***PT*-related hinge states under various geometries. a-d** Plots of the hinge state distribution for different geometries in the xz plane. In each plot, the red circles correspond to the sum of the probabilities of all hinge states and the grey background indicates the shape of the lattice. The lattices have tubelike geometries, with periodic boundary condition along the y direction and open boundary condition along the x and z directions. The coupling parameters used in the calculations are: $t = 1, J_1^x = 1, J_2^x = 2.2, J_1^z = 1, J_2^z = 1.6$.

the y direction and open boundary condition along the x and z directions). The first two plots (Fig. S8a, b) correspond to the two samples demonstrated in the main text. We can clearly see one and three pairs of PT-related hinge states in the respective plot, which also agree with our simulations and experiments. Fig. S8c, d give another two examples under different geometries. In particular, Fig. S8d shows that the PT-related hinge states can also occur at the inner hinges of the lattice. We note that the coupling parameters remain the same for all calculations, which indicates that the number and the position of the hinge states are highly tunable by engineering the lattice shape, even without the need to change lattice parameters.

* yuxinphy@hku.hk

† blzhang@ntu.edu.sg

[1] Y. X. Zhao, A. P. Schnyder, and Z. D. Wang, Unified theory of PT and CP invariant topological metals and nodal superconductors, Phys. Rev. Lett. **116**, 156402 (2016).

[2] M. Nakahara, Geometry, topology and physics (CRC press, 2018).

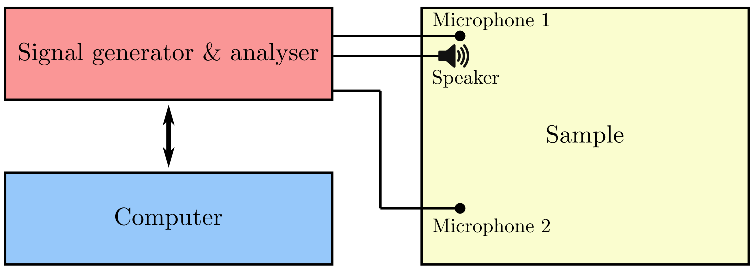

Fig. S9. **Schematic of the experimental setup.** In the experiments, the properties of the excitation signal (i.e., amplitude, frequency range, etc.) and the processing process (i.e., average time, data collecting frequencies, etc.) are set in the computer and passed to the signal generator and analyser. Then, a speaker connected to the signal generator and analyser launches the sound signal into the sample accordingly. Next, the acoustic field distribution in the sample is measured by the microphones. Here, microphone 1 is placed next to the speaker, working as the reference probe. Microphone 2 is the scanning probe and measures the field distribution. Finally, the measured signal is processed by the signal generator and analyser and the processed data are sent to the computer for plotting and further analysis.

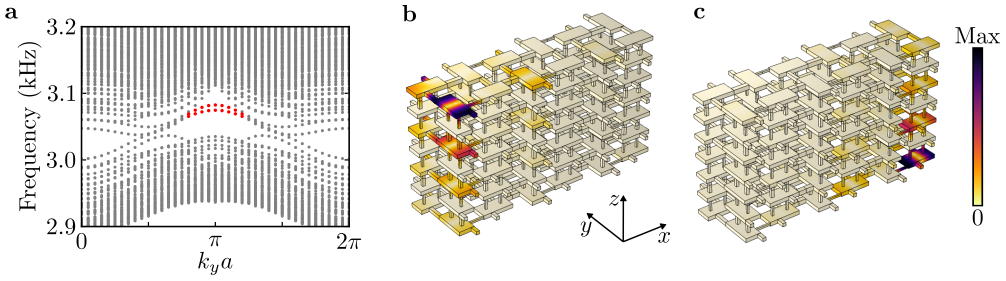

Fig. S10. **Diagonal hinge states by reversing the z-dimensional dimerization.** **a**, Simulated dispersion for a structure similar to the one in Fig. 4 in the main text but with swapped d_{z1} and d_{z2}. The grey dots represent the bulk and SSs, and the red dots indicate the hinge bands. **b-c**, Eigen profiles for the two HSs at $k_y = \pi/a$, showing the diagonal distribution of the HSs. The color indicates the amplitude of the acoustic pressure. The corresponding eigenfrequencies are 3072.3 Hz (**b**) and 3080.8 Hz (**c**). In **a**, for a clear visualization of the dispersion, we use 16 and 17 cavities along the x and z directions, respectively. In **b-c**, we use 6 and 7 cavities along the x and z directions, respectively. Periodic boundary condition is imposed to the y direction for both simulations.

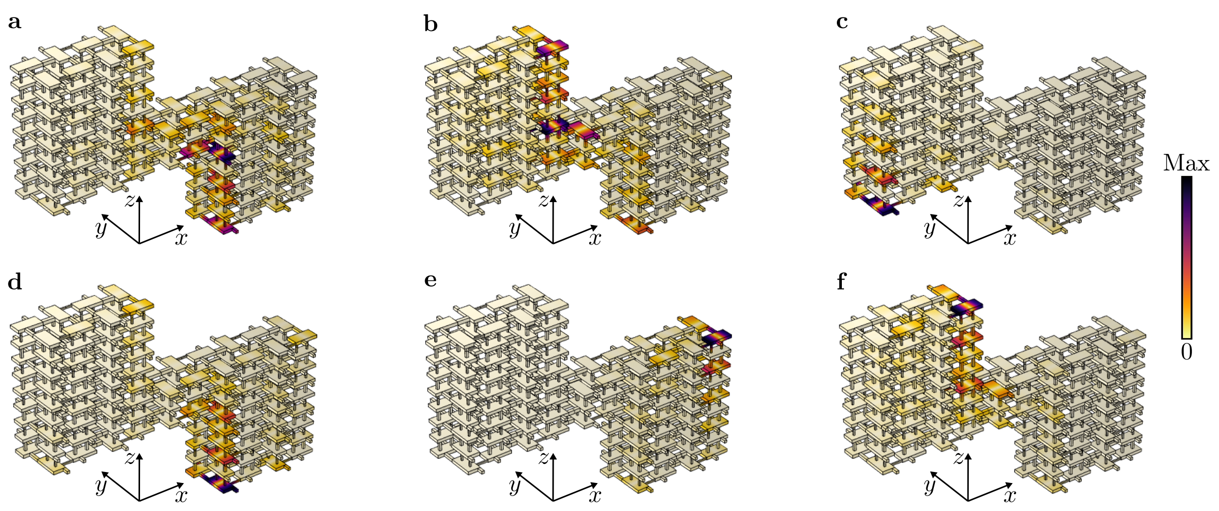

Fig. S11. **Eigen profiles of the six states highlighted in red in Fig. 5c.** The color indicates the amplitude of the acoustic pressure. The corresponding eigenfrequencies are 3058.3 Hz (**a**), 3067.4 Hz (**b**), 3072.6 Hz (**c**), 3076.4 Hz (**d**), 3080.6 Hz (**e**) and 3084.8 Hz (**f**). These states are localized around the six hinges denoted by the red circles in Fig. 5b in the main text. In particular, the HSs at the two obtuse-angled hinges feature a "off-center" field pattern (see **a** and **b**), consistent with the measurements shown in Fig. S13c, g.

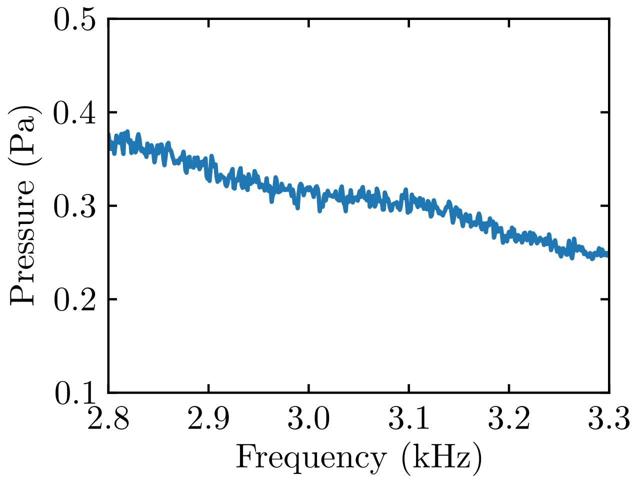

Fig. S12. **Measured acoustic pressure spectrum of the source.**

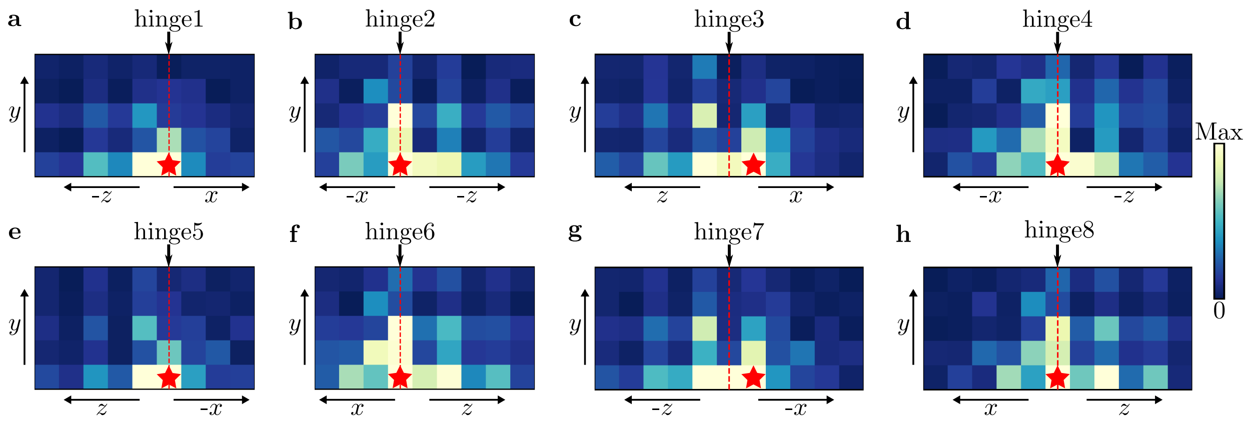

Fig. S13. **Measured acoustic field distributions for the sample shown in Fig. 5a. a–h**, Experimentally measured acoustic intensity distributions on two surfaces adjacent to hinge 1 (**a**), hinge 2 (**b**), hinge 3 (**c**), hinge 4 (**d**), hinge 5 (**e**), hinge 6 (**f**), hinge 7 (**g**), hinge 8 (**h**). The red star indicates the position of the speaker and the red dashed line highlights the position of the hinge. The operating frequencies of the speaker are chosen as: 3075 Hz (hinge 1 and hinge 5), 3076 Hz (hinge 2), 3062 Hz (hinge 3) and 3081 Hz (hinge 4), 3078 Hz (hinge 6), 3051 Hz (hinge 7) and 3067 Hz (hinge 8), which are around the eigenfrequencies of the HSs.
